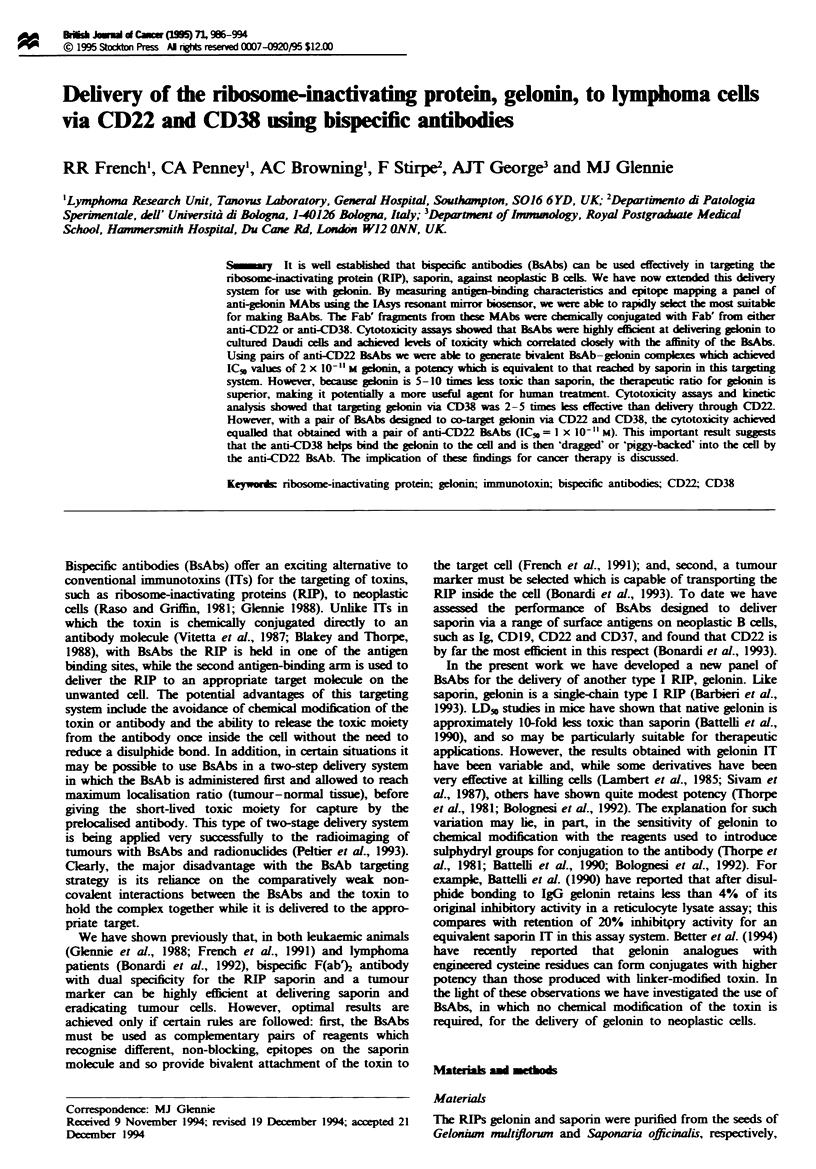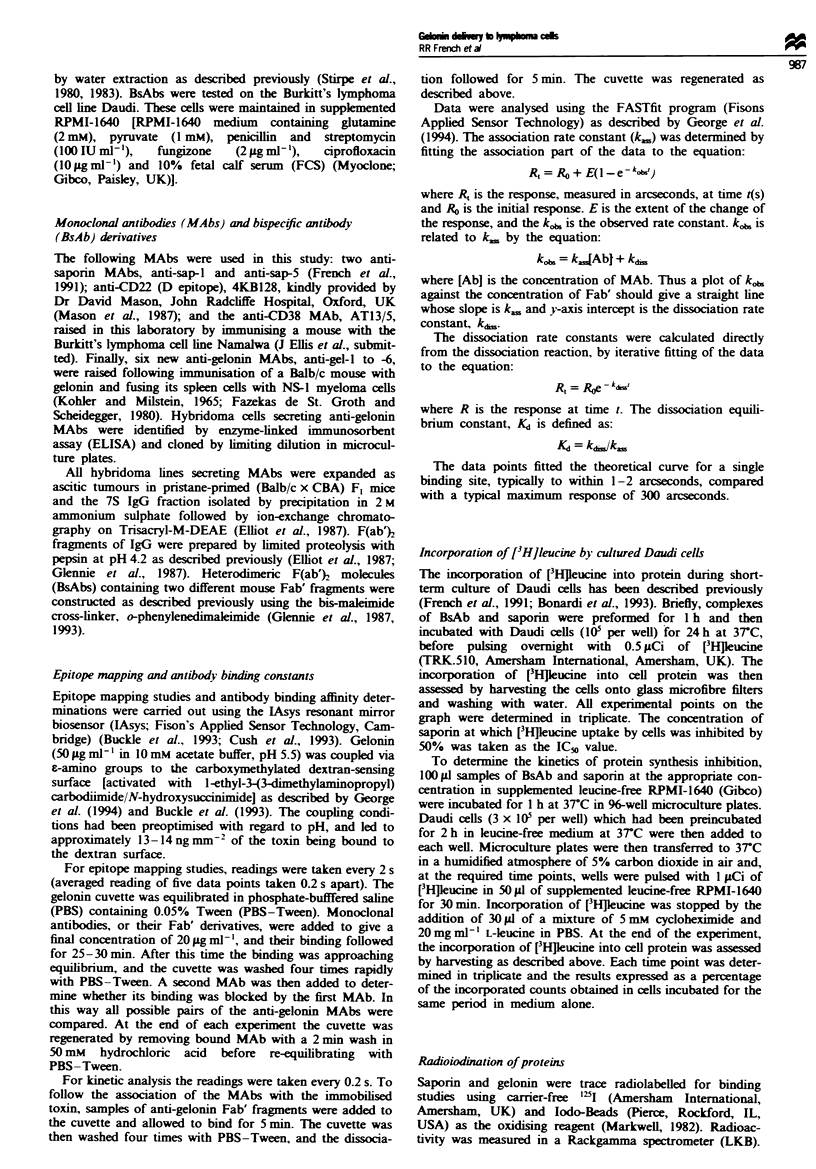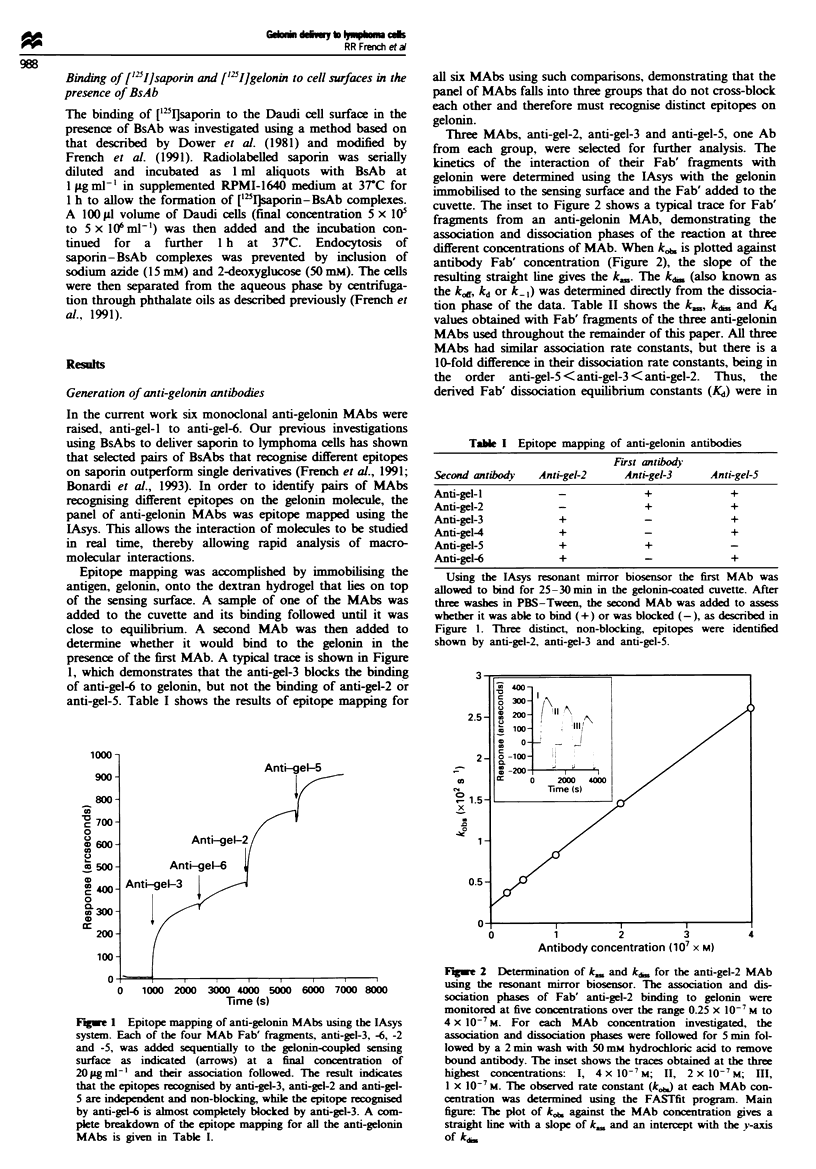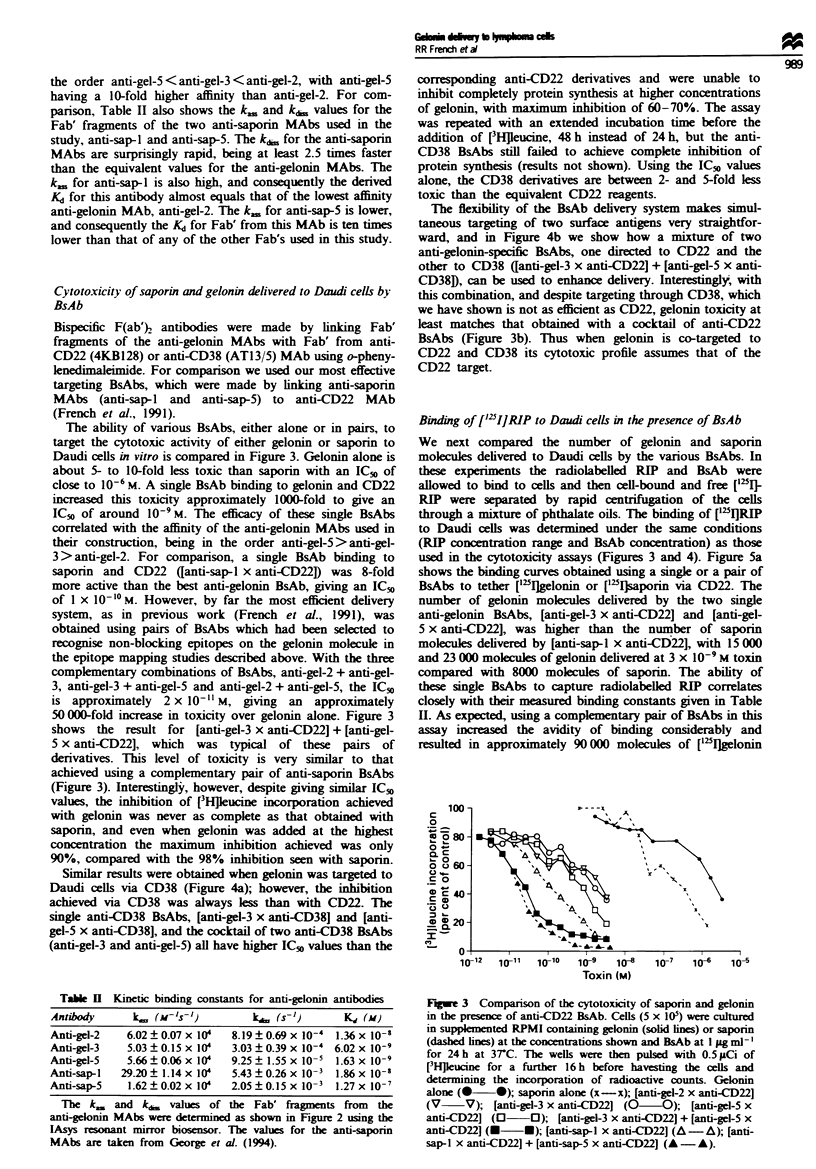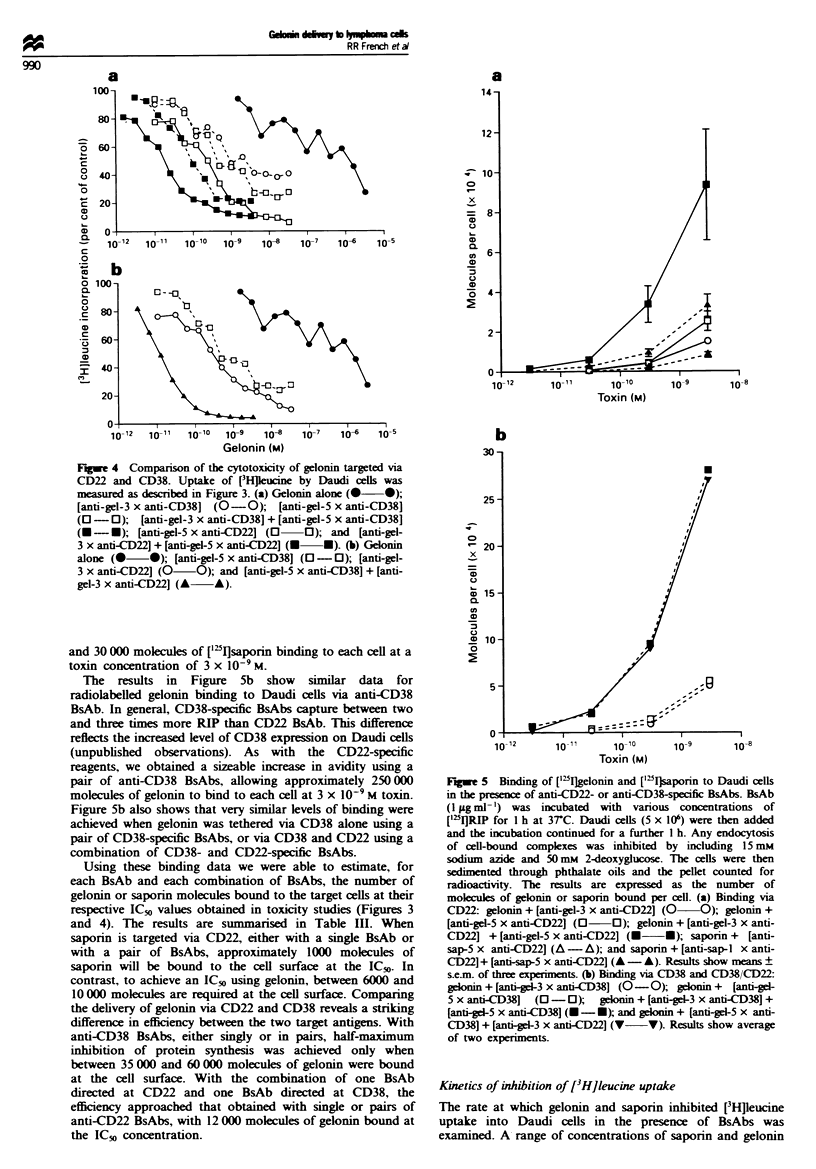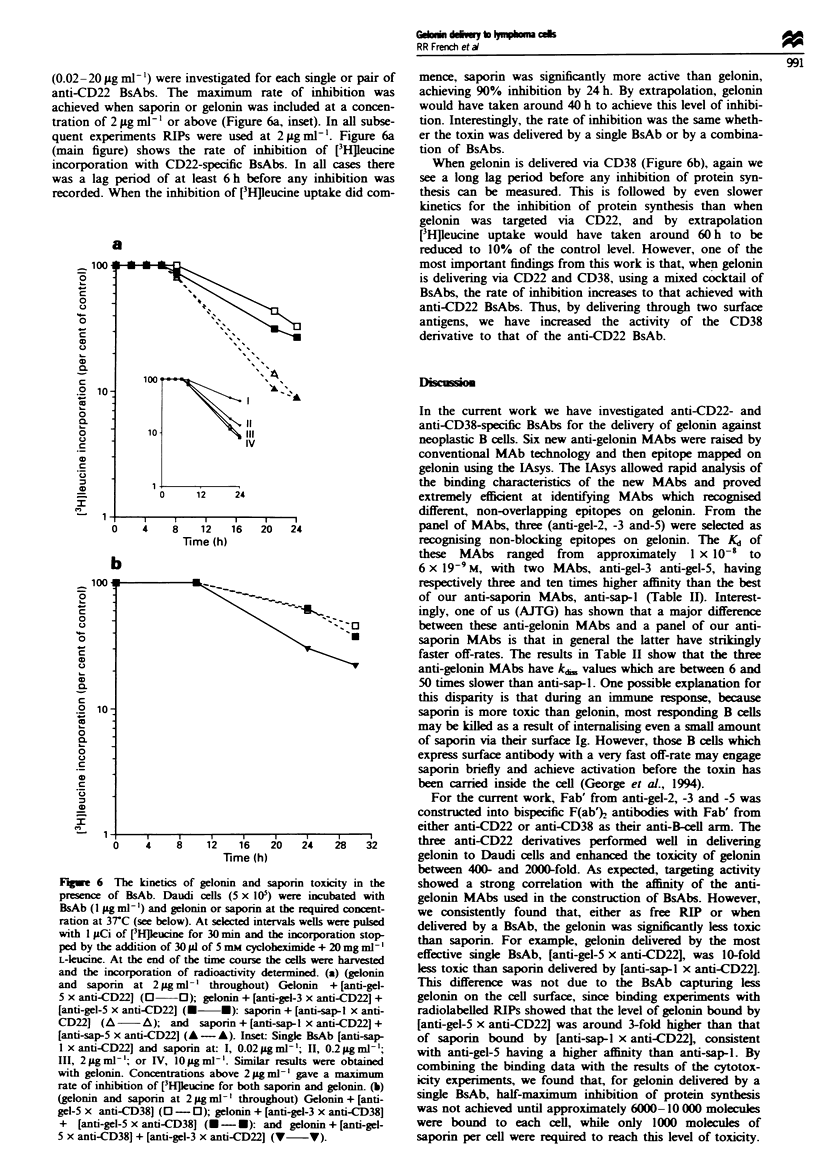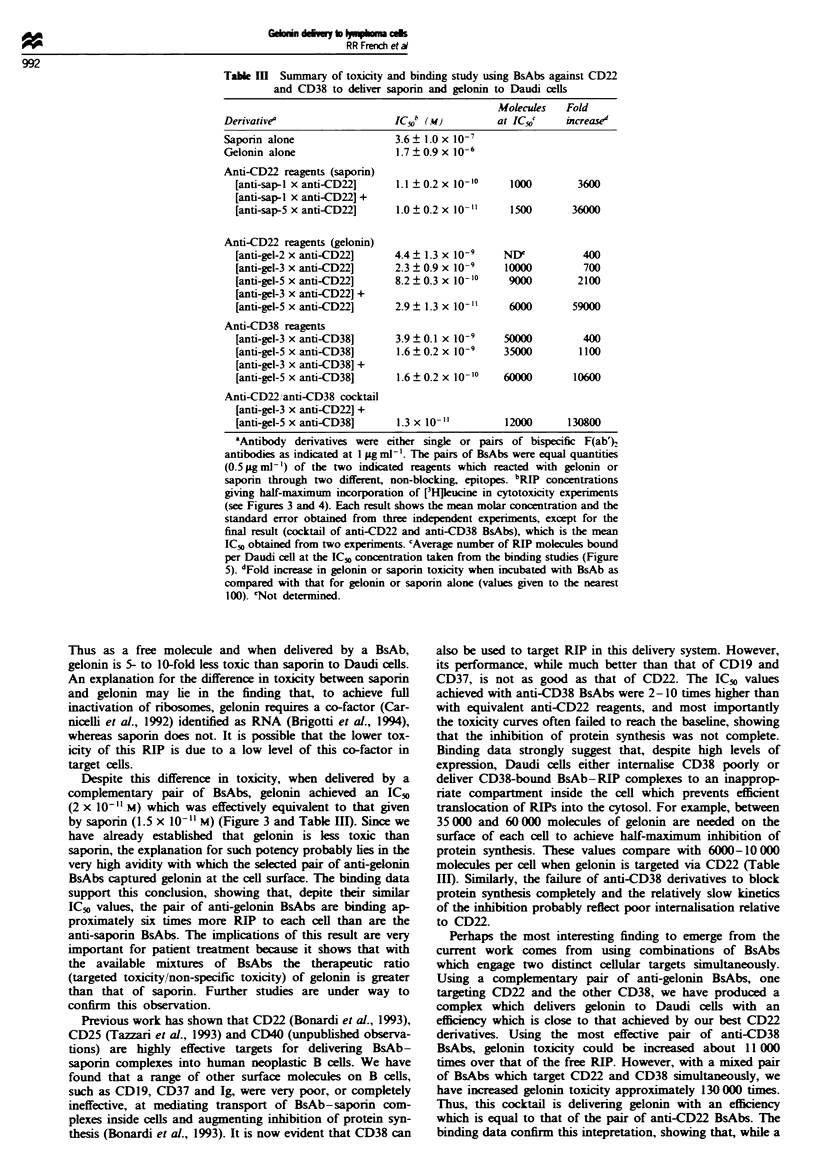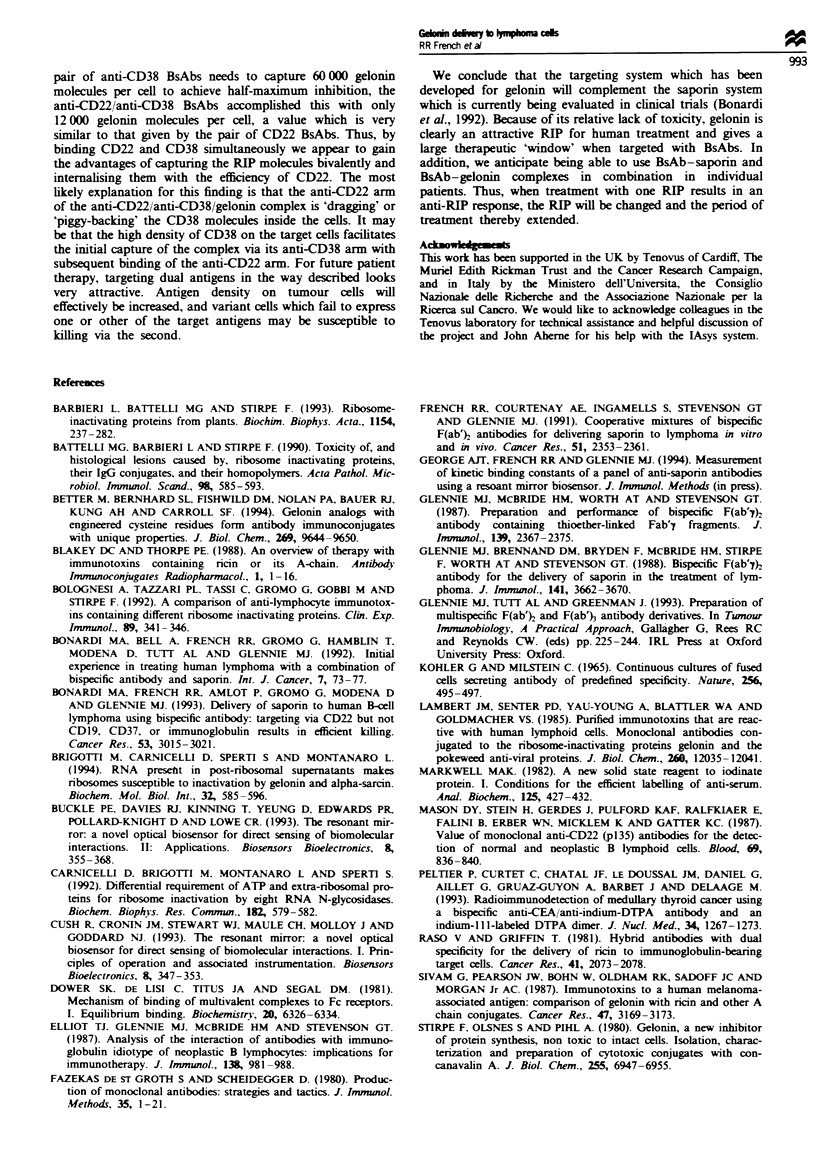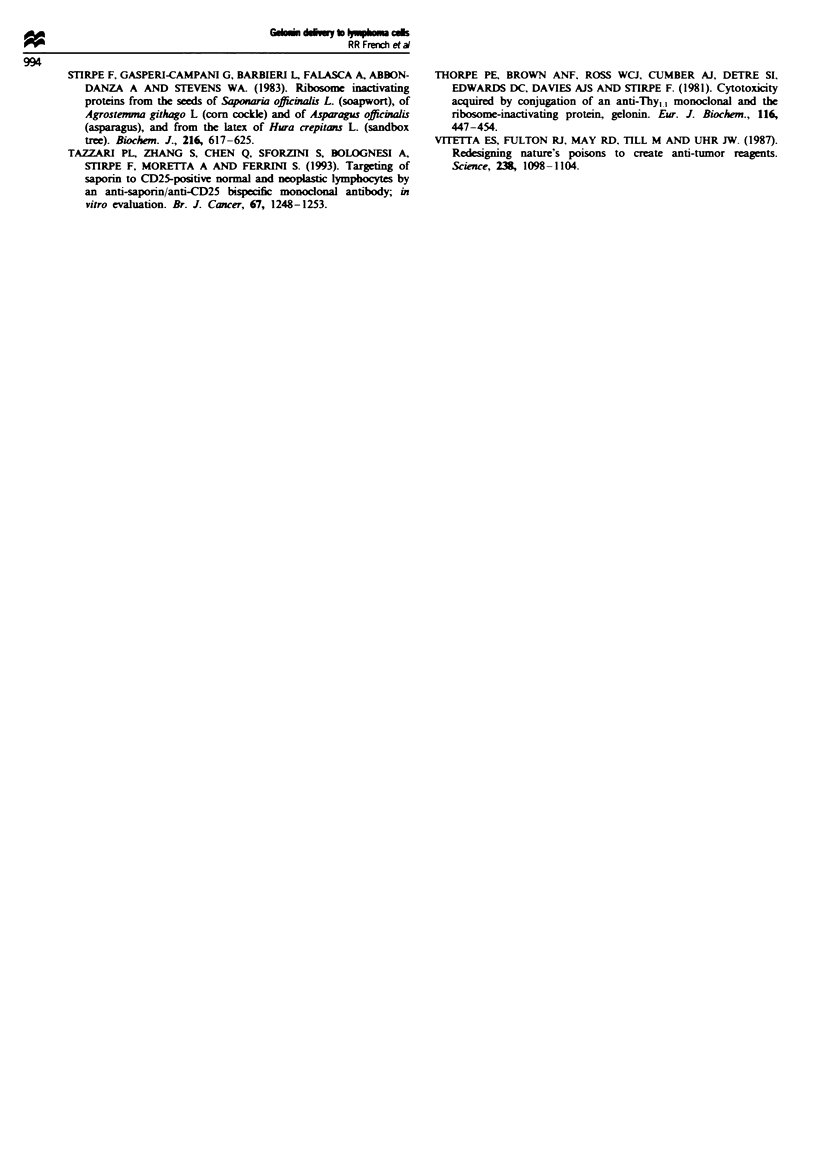# Delivery of the ribosome-inactivating protein, gelonin, to lymphoma cells via CD22 and CD38 using bispecific antibodies.

**DOI:** 10.1038/bjc.1995.190

**Published:** 1995-05

**Authors:** R. R. French, C. A. Penney, A. C. Browning, F. Stirpe, A. J. George, M. J. Glennie

**Affiliations:** Lymphoma Research Unit, Tanovus Laboratory, General Hospital, Southampton, UK.

## Abstract

It is well established that bispecific antibodies (BsAbs) can be used effectively in targeting the ribosome-inactivating protein (RIP), saporin, against neoplastic B cells. We have now extended this delivery system for use with gelonin. By measuring antigen-binding characteristics and epitope mapping a panel of anti-gelonin MAbs using the IAsys resonant mirror bisensor, we were able to rapidly select the most suitable for making BaAbs. The Fab' fragments from these MAbs were chemically conjugated with Fab' from either anti-CD22 or anti-CD38. Cytotoxicity assays showed that BsAbs were highly efficient at delivering gelonin to cultured Daudi cells and achieved levels of toxicity which correlated closely with the affinity of the BsAbs. Using pairs of anti-CD22 BsAbs we were able to generate bivalent BsAb-gelonin complexes which achieved IC50 values of 2 x 10(-11) M gelonin, a potency which is equivalent to that reached by saporin in this targeting system. However, because gelonin is 5-10 times less toxic than saporin, the therapeutic ratio for gelonin is superior, making it potentially a more useful agent for human treatment. Cytotoxicity assays and kinetic analysis showed that targeting gelonin via CD38 was 2-5 times less effective than delivery through CD22. However, with a pair of BsAbs designed to co-target gelonin via CD22 and CD38, the cytotoxicity achieved equalled that obtained with a pair of anti-CD22 BsAbs (IC50 = 1 x 10(-11) M). This important result suggests that the anti-CD38 helps bind the gelonin to the cell and is then 'dragged' or 'piggy-backed' into the cell by the anti-CD22 BsAb. The implication of these findings for cancer therapy is discussed.


© 1995 Stockton Press All rights reserved 0007-0920/95 $12.00

### Delivery of the ribosome-inactivating protein, gelonin, to lymphoma cells via CD22 and CD38 using bispecific antibodies

RR French[1], CA Penney[1], AC Browning[1], F Stirpe[2], AJT George[3] and MJ Glennie

[1]*Lymphoma Research Unit, Tanovus Laboratory, General Hospital, Southampton, SO16 6YD, UK;* [2]*Departimento di Patologia Sperimentale, dell' Università di Bologna, I-40126 Bologna, Italy;* [3]*Department of Immunology, Royal Postgraduate Medical School, Hammersmith Hospital, Du Cane Rd, London W12 0NN, UK.*

**Summary** It is well established that bispecific antibodies (BsAbs) can be used effectively in targeting the ribosome-inactivating protein (RIP), saporin, against neoplastic B cells. We have now extended this delivery system for use with gelonin. By measuring antigen-binding characteristics and epitope mapping a panel of anti-gelonin MAbs using the IAsys resonant mirror biosensor, we were able to rapidly select the most suitable for making BaAbs. The Fab' fragments from these MAbs were chemically conjugated with Fab' from either anti-CD22 or anti-CD38. Cytotoxicity assays showed that BsAbs were highly efficient at delivering gelonin to cultured Daudi cells and achieved levels of toxicity which correlated closely with the affinity of the BsAbs. Using pairs of anti-CD22 BsAbs we were able to generate bivalent BsAb-gelonin complexes which achieved $IC_{50}$ values of $2 \times 10^{-11}$ M gelonin, a potency which is equivalent to that reached by saporin in this targeting system. However, because gelonin is 5-10 times less toxic than saporin, the therapeutic ratio for gelonin is superior, making it potentially a more useful agent for human treatment. Cytotoxicity assays and kinetic analysis showed that targeting gelonin via CD38 was 2-5 times less effective than delivery through CD22. However, with a pair of BsAbs designed to co-target gelonin via CD22 and CD38, the cytotoxicity achieved equalled that obtained with a pair of anti-CD22 BsAbs ($IC_{50} = 1 \times 10^{-11}$ M). This important result suggests that the anti-CD38 helps bind the gelonin to the cell and is then 'dragged' or 'piggy-backed' into the cell by the anti-CD22 BsAb. The implication of these findings for cancer therapy is discussed.

**Keywords:** ribosome-inactivating protein; gelonin; immunotoxin; bispecific antibodies; CD22; CD38

Bispecific antibodies (BsAbs) offer an exciting alternative to conventional immunotoxins (ITs) for the targeting of toxins, such as ribosome-inactivating proteins (RIP), to neoplastic cells (Raso and Griffin, 1981; Glennie 1988). Unlike ITs in which the toxin is chemically conjugated directly to an antibody molecule (Vitetta *et al*., 1987; Blakey and Thorpe, 1988), with BsAbs the RIP is held in one of the antigen binding sites, while the second antigen-binding arm is used to deliver the RIP to an appropriate target molecule on the unwanted cell. The potential advantages of this targeting system include the avoidance of chemical modification of the toxin or antibody and the ability to release the toxic moiety from the antibody once inside the cell without the need to reduce a disulphide bond. In addition, in certain situations it may be possible to use BsAbs in a two-step delivery system in which the BsAb is administered first and allowed to reach maximum localisation ratio (tumour-normal tissue), before giving the short-lived toxic moiety for capture by the prelocalised antibody. This type of two-stage delivery system is being applied very successfully to the radioimaging of tumours with BsAbs and radionuclides (Peltier *et al*., 1993). Clearly, the major disadvantage with the BsAb targeting strategy is its reliance on the comparatively weak non-covalent interactions between the BsAbs and the toxin to hold the complex together while it is delivered to the appropriate target.

We have shown previously that, in both leukaemic animals (Glennie *et al*., 1988; French *et al*., 1991) and lymphoma patients (Bonardi *et al*., 1992), bispecific F(ab')$_2$ antibody with dual specificity for the RIP saporin and a tumour marker can be highly efficient at delivering saporin and eradicating tumour cells. However, optimal results are achieved only if certain rules are followed: first, the BsAbs must be used as complementary pairs of reagents which recognise different, non-blocking, epitopes on the saporin molecule and so provide bivalent attachment of the toxin to the target cell (French *et al*., 1991); and, second, a tumour marker must be selected which is capable of transporting the RIP inside the cell (Bonardi *et al*., 1993). To date we have assessed the performance of BsAbs designed to deliver saporin via a range of surface antigens on neoplastic B cells, such as Ig, CD19, CD22 and CD37, and found that CD22 is by far the most efficient in this respect (Bonardi *et al*., 1993).

In the present work we have developed a new panel of BsAbs for the delivery of another type I RIP, gelonin. Like saporin, gelonin is a single-chain type I RIP (Barbieri *et al*., 1993). $LD_{50}$ studies in mice have shown that native gelonin is approximately 10-fold less toxic than saporin (Battelli *et al*., 1990), and so may be particularly suitable for therapeutic applications. However, the results obtained with gelonin IT have been variable and, while some derivatives have been very effective at killing cells (Lambert *et al*., 1985; Sivam *et al*., 1987), others have shown quite modest potency (Thorpe *et al*., 1981; Bolognesi *et al*., 1992). The explanation for such variation may lie, in part, in the sensitivity of gelonin to chemical modification with the reagents used to introduce sulphydryl groups for conjugation to the antibody (Thorpe *et al*., 1981; Battelli *et al*., 1990; Bolognesi *et al*., 1992). For example, Battelli *et al*. (1990) have reported that after disulphide bonding to IgG gelonin retains less than 4% of its original inhibitory activity in a reticulocyte lysate assay; this compares with retention of 20% inhibitory activity for an equivalent saporin IT in this assay system. Better *et al*. (1994) have recently reported that gelonin analogues with engineered cysteine residues can form conjugates with higher potency than those produced with linker-modified toxin. In the light of these observations we have investigated the use of BsAbs, in which no chemical modification of the toxin is required, for the delivery of gelonin to neoplastic cells.

### Materials and methods

### *Materials*

The RIPs gelonin and saporin were purified from the seeds of *Gelonium multiflorum* and *Saponaria officinalis*, respectively,

Correspondence: MJ Glennie
Received 9 November 1994; revised 19 December 1994; accepted 21 December 1994

by water extraction as described previously (Stirpe et al., 1980, 1983). BsAbs were tested on the Burkitt's lymphoma cell line Daudi. These cells were maintained in supplemented RPMI-1640 [RPMI-1640 medium containing glutamine (2 mM), pyruvate (1 mM), penicillin and streptomycin ($100\ IU\ ml^{-1}$), fungizone ($2\ \mu g\ ml^{-1}$), ciprofloxacin ($10\ \mu g\ ml^{-1}$) and 10% fetal calf serum (FCS) (Myoclone; Gibco, Paisley, UK)].

### *Monoclonal antibodies (MAbs) and bispecific antibody (BsAb) derivatives*

The following MAbs were used in this study: two anti-saporin MAbs, anti-sap-1 and anti-sap-5 (French et al., 1991); anti-CD22 (D epitope), 4KB128, kindly provided by Dr David Mason, John Radcliffe Hospital, Oxford, UK (Mason et al., 1987); and the anti-CD38 MAb, AT13/5, raised in this laboratory by immunising a mouse with the Burkitt's lymphoma cell line Namalwa (J Ellis et al., submitted). Finally, six new anti-gelonin MAbs, anti-gel-1 to -6, were raised following immunisation of a Balb/c mouse with gelonin and fusing its spleen cells with NS-1 myeloma cells (Kohler and Milstein, 1965; Fazekas de St. Groth and Scheidegger, 1980). Hybridoma cells secreting anti-gelonin MAbs were identified by enzyme-linked immunosorbent assay (ELISA) and cloned by limiting dilution in microculture plates.

All hybridoma lines secreting MAbs were expanded as ascitic tumours in pristane-primed (Balb/c × CBA) $F_1$ mice and the 7S IgG fraction isolated by precipitation in 2 M ammonium sulphate followed by ion-exchange chromatography on Trisacryl-M-DEAE (Elliot et al., 1987). $F(ab')_2$ fragments of IgG were prepared by limited proteolysis with pepsin at pH 4.2 as described previously (Elliot et al., 1987; Glennie et al., 1987). Heterodimeric $F(ab')_2$ molecules (BsAbs) containing two different mouse Fab' fragments were constructed as described previously using the bis-maleimide cross-linker, o-phenylenedimaleimide (Glennie et al., 1987, 1993).

### *Epitope mapping and antibody binding constants*

Epitope mapping studies and antibody binding affinity determinations were carried out using the IAsys resonant mirror biosensor (IAsys; Fison's Applied Sensor Technology, Cambridge) (Buckle et al., 1993; Cush et al., 1993). Gelonin ($50\ \mu g\ ml^{-1}$ in 10 mM acetate buffer, pH 5.5) was coupled via ε-amino groups to the carboxymethylated dextran-sensing surface [activated with 1-ethyl-3-(3-dimethylaminopropyl) carbodiimide/N-hydroxysuccinimide] as described by George et al. (1994) and Buckle et al. (1993). The coupling conditions had been preoptimised with regard to pH, and led to approximately 13–14 $ng\ mm^{-2}$ of the toxin being bound to the dextran surface.

For epitope mapping studies, readings were taken every 2 s (averaged reading of five data points taken 0.2 s apart). The gelonin cuvette was equilibrated in phosphate-bufffered saline (PBS) containing 0.05% Tween (PBS–Tween). Monoclonal antibodies, or their Fab' derivatives, were added to give a final concentration of $20\ \mu g\ ml^{-1}$, and their binding followed for 25–30 min. After this time the binding was approaching equilibrium, and the cuvette was washed four times rapidly with PBS–Tween. A second MAb was then added to determine whether its binding was blocked by the first MAb. In this way all possible pairs of the anti-gelonin MAbs were compared. At the end of each experiment the cuvette was regenerated by removing bound MAb with a 2 min wash in 50 mM hydrochloric acid before re-equilibrating with PBS–Tween.

For kinetic analysis the readings were taken every 0.2 s. To follow the association of the MAbs with the immobilised toxin, samples of anti-gelonin Fab' fragments were added to the cuvette and allowed to bind for 5 min. The cuvette was then washed four times with PBS–Tween, and the dissociation followed for 5 min. The cuvette was regenerated as described above.

Data were analysed using the FASTfit program (Fisons Applied Sensor Technology) as described by George et al. (1994). The association rate constant ($k_{ass}$) was determined by fitting the association part of the data to the equation:

$$R_t = R_0 + E(1 - e^{-k_{obs}t})$$

where $R_t$ is the response, measured in arcseconds, at time $t$(s) and $R_0$ is the initial response. $E$ is the extent of the change of the response, and the $k_{obs}$ is the observed rate constant. $k_{obs}$ is related to $k_{ass}$ by the equation:

$$k_{obs} = k_{ass}[Ab] + k_{diss}$$

where [Ab] is the concentration of MAb. Thus a plot of $k_{obs}$ against the concentration of Fab' should give a straight line whose slope is $k_{ass}$ and $y$-axis intercept is the dissociation rate constant, $k_{diss}$.

The dissociation rate constants were calculated directly from the dissociation reaction, by iterative fitting of the data to the equation:

$$R_t = R_0 e^{-k_{diss}t}$$

where $R$ is the response at time $t$. The dissociation equilibrium constant, $K_d$ is defined as:

$$K_d = k_{diss}/k_{ass}$$

The data points fitted the theoretical curve for a single binding site, typically to within 1–2 arcseconds, compared with a typical maximum response of 300 arcseconds.

### *Incorporation of [³H]leucine by cultured Daudi cells*

The incorporation of [$^3$H]leucine into protein during short-term culture of Daudi cells has been described previously (French et al., 1991; Bonardi et al., 1993). Briefly, complexes of BsAb and saporin were preformed for 1 h and then incubated with Daudi cells ($10^5$ per well) for 24 h at 37°C, before pulsing overnight with 0.5 μCi of [$^3$H]leucine (TRK.510, Amersham International, Amersham, UK). The incorporation of [$^3$H]leucine into cell protein was then assessed by harvesting the cells onto glass microfibre filters and washing with water. All experimental points on the graph were determined in triplicate. The concentration of saporin at which [$^3$H]leucine uptake by cells was inhibited by 50% was taken as the $IC_{50}$ value.

To determine the kinetics of protein synthesis inhibition, 100 μl samples of BsAb and saporin at the appropriate concentration in supplemented leucine-free RPMI-1640 (Gibco) were incubated for 1 h at 37°C in 96-well microculture plates. Daudi cells ($3 \times 10^5$ per well) which had been preincubated for 2 h in leucine-free medium at 37°C were then added to each well. Microculture plates were then transferred to 37°C in a humidified atmosphere of 5% carbon dioxide in air and, at the required time points, wells were pulsed with 1 μCi of [$^3$H]leucine in 50 μl of supplemented leucine-free RPMI-1640 for 30 min. Incorporation of [$^3$H]leucine was stopped by the addition of 30 μl of a mixture of 5 mM cycloheximide and $20\ mg\ ml^{-1}$ L-leucine in PBS. At the end of the experiment, the incorporation of [$^3$H]leucine into cell protein was assessed by harvesting as described above. Each time point was determined in triplicate and the results expressed as a percentage of the incorporated counts obtained in cells incubated for the same period in medium alone.

### *Radioiodination of proteins*

Saporin and gelonin were trace radiolabelled for binding studies using carrier-free $^{125}$I (Amersham International, Amersham, UK) and Iodo-Beads (Pierce, Rockford, IL, USA) as the oxidising reagent (Markwell, 1982). Radioactivity was measured in a Rackgamma spectrometer (LKB).

*Binding of [$^{125}$I]saporin and [$^{125}$I]gelonin to cell surfaces in the presence of BsAb*

The binding of [$^{125}$I]saporin to the Daudi cell surface in the presence of BsAb was investigated using a method based on that described by Dower *et al.* (1981) and modified by French *et al.* (1991). Radiolabelled saporin was serially diluted and incubated as 1 ml aliquots with BsAb at 1 $\mu$g ml$^{-1}$ in supplemented RPMI-1640 medium at 37°C for 1 h to allow the formation of [$^{125}$I]saporin–BsAb complexes. A 100 $\mu$l volume of Daudi cells (final concentration $5 \times 10^5$ to $5 \times 10^6$ ml$^{-1}$) was then added and the incubation continued for a further 1 h at 37°C. Endocytosis of saporin–BsAb complexes was prevented by inclusion of sodium azide (15 mM) and 2-deoxyglucose (50 mM). The cells were then separated from the aqueous phase by centrifugation through phthalate oils as described previously (French *et al.*, 1991).

### Results

*Generation of anti-gelonin antibodies*

In the current work six monoclonal anti-gelonin MAbs were raised, anti-gel-1 to anti-gel-6. Our previous investigations using BsAbs to deliver saporin to lymphoma cells has shown that selected pairs of BsAbs that recognise different epitopes on saporin outperform single derivatives (French *et al.*, 1991; Bonardi *et al.*, 1993). In order to identify pairs of MAbs recognising different epitopes on the gelonin molecule, the panel of anti-gelonin MAbs was epitope mapped using the IAsys. This allows the interaction of molecules to be studied in real time, thereby allowing rapid analysis of macromolecular interactions.

Epitope mapping was accomplished by immobilising the antigen, gelonin, onto the dextran hydrogel that lies on top of the sensing surface. A sample of one of the MAbs was added to the cuvette and its binding followed until it was close to equilibrium. A second MAb was then added to determine whether it would bind to the gelonin in the presence of the first MAb. A typical trace is shown in Figure 1, which demonstrates that the anti-gel-3 blocks the binding of anti-gel-6 to gelonin, but not the binding of anti-gel-2 or anti-gel-5. Table I shows the results of epitope mapping for all six MAbs using such comparisons, demonstrating that the panel of MAbs falls into three groups that do not cross-block each other and therefore must recognise distinct epitopes on gelonin.

Three MAbs, anti-gel-2, anti-gel-3 and anti-gel-5, one Ab from each group, were selected for further analysis. The kinetics of the interaction of their Fab′ fragments with gelonin were determined using the IAsys with the gelonin immobilised to the sensing surface and the Fab′ added to the cuvette. The inset to Figure 2 shows a typical trace for Fab′ fragments from an anti-gelonin MAb, demonstrating the association and dissociation phases of the reaction at three different concentrations of MAb. When $k_{obs}$ is plotted against antibody Fab′ concentration (Figure 2), the slope of the resulting straight line gives the $k_{ass}$. The $k_{diss}$ (also known as the $k_{off}$, $k_d$ or $k_{-1}$) was determined directly from the dissociation phase of the data. Table II shows the $k_{ass}$, $k_{diss}$ and $K_d$ values obtained with Fab′ fragments of the three anti-gelonin MAbs used throughout the remainder of this paper. All three MAbs had similar association rate constants, but there is a 10-fold difference in their dissociation rate constants, being in the order anti-gel-5 < anti-gel-3 < anti-gel-2. Thus, the derived Fab′ dissociation equilibrium constants ($K_d$) were in

**Table I** Epitope mapping of anti-gelonin antibodies

| | *First antibody* | | |
|---|---|---|---|
| *Second antibody* | *Anti-gel-2* | *Anti-gel-3* | *Anti-gel-5* |
| Anti-gel-1 | – | + | + |
| Anti-gel-2 | – | + | + |
| Anti-gel-3 | + | – | + |
| Anti-gel-4 | + | – | + |
| Anti-gel-5 | + | + | – |
| Anti-gel-6 | + | – | + |

Using the IAsys resonant mirror biosensor the first MAb was allowed to bind for 25–30 min in the gelonin-coated cuvette. After three washes in PBS–Tween, the second MAb was added to assess whether it was able to bind (+) or was blocked (–), as described in Figure 1. Three distinct, non-blocking, epitopes were identified shown by anti-gel-2, anti-gel-3 and anti-gel-5.

**Figure 1** Epitope mapping of anti-gelonin MAbs using the IAsys system. Each of the four MAb Fab′ fragments, anti-gel-3, -6, -2 and -5, was added sequentially to the gelonin-coupled sensing surface as indicated (arrows) at a final concentration of 20 $\mu$g ml$^{-1}$ and their association followed. The result indicates that the epitopes recognised by anti-gel-3, anti-gel-2 and anti-gel-5 are independent and non-blocking, while the epitope recognised by anti-gel-6 is almost completely blocked by anti-gel-3. A complete breakdown of the epitope mapping for all the anti-gelonin MAbs is given in Table I.

**Figure 2** Determination of $k_{ass}$ and $k_{diss}$ for the anti-gel-2 MAb using the resonant mirror biosensor. The association and dissociation phases of Fab′ anti-gel-2 binding to gelonin were monitored at five concentrations over the range $0.25 \times 10^{-7}$ M to $4 \times 10^{-7}$ M. For each MAb concentration investigated, the association and dissociation phases were followed for 5 min followed by a 2 min wash with 50 mM hydrochloric acid to remove bound antibody. The inset shows the traces obtained at the three highest concentrations: I, $4 \times 10^{-7}$ M; II, $2 \times 10^{-7}$ M; III, $1 \times 10^{-7}$ M. The observed rate constant ($k_{obs}$) at each MAb concentration was determined using the FASTfit program. Main figure: The plot of $k_{obs}$ against the MAb concentration gives a straight line with a slope of $k_{ass}$ and an intercept with the *y*-axis of $k_{diss}$

the order anti-gel-5 < anti-gel-3 < anti-gel-2, with anti-gel-5 having a 10-fold higher affinity than anti-gel-2. For comparison, Table II also shows the $k_{ass}$ and $k_{diss}$ values for the Fab′ fragments of the two anti-saporin MAbs used in the study, anti-sap-1 and anti-sap-5. The $k_{diss}$ for the anti-saporin MAbs are surprisingly rapid, being at least 2.5 times faster than the equivalent values for the anti-gelonin MAbs. The $k_{ass}$ for anti-sap-1 is also high, and consequently the derived $K_d$ for this antibody almost equals that of the lowest affinity anti-gelonin MAb, anti-gel-2. The $k_{ass}$ for anti-sap-5 is lower, and consequently the $K_d$ for Fab′ from this MAb is ten times lower than that of any of the other Fab′s used in this study.

### *Cytotoxicity of saporin and gelonin delivered to Daudi cells by BsAb*

Bispecific $F(ab')_2$ antibodies were made by linking Fab′ fragments of the anti-gelonin MAbs with Fab′ from anti-CD22 (4KB128) or anti-CD38 (AT13/5) MAb using *o*-phenylenedimaleimide. For comparison we used our most effective targeting BsAbs, which were made by linking anti-saporin MAbs (anti-sap-1 and anti-sap-5) to anti-CD22 MAb (French *et al.*, 1991).

The ability of various BsAbs, either alone or in pairs, to target the cytotoxic activity of either gelonin or saporin to Daudi cells *in vitro* is compared in Figure 3. Gelonin alone is about 5- to 10-fold less toxic than saporin with an $IC_{50}$ of close to $10^{-6}$ M. A single BsAb binding to gelonin and CD22 increased this toxicity approximately 1000-fold to give an $IC_{50}$ of around $10^{-9}$ M. The efficacy of these single BsAbs correlated with the affinity of the anti-gelonin MAbs used in their construction, being in the order anti-gel-5 > anti-gel-3 > anti-gel-2. For comparison, a single BsAb binding to saporin and CD22 ([anti-sap-1 × anti-CD22]) was 8-fold more active than the best anti-gelonin BsAb, giving an $IC_{50}$ of $1 \times 10^{-10}$ M. However, by far the most efficient delivery system, as in previous work (French *et al.*, 1991), was obtained using pairs of BsAbs which had been selected to recognise non-blocking epitopes on the gelonin molecule in the epitope mapping studies described above. With the three complementary combinations of BsAbs, anti-gel-2 + anti-gel-3, anti-gel-3 + anti-gel-5 and anti-gel-2 + anti-gel-5, the $IC_{50}$ is approximately $2 \times 10^{-11}$ M, giving an approximately 50 000-fold increase in toxicity over gelonin alone. Figure 3 shows the result for [anti-gel-3 × anti-CD22] + [anti-gel-5 × anti-CD22], which was typical of these pairs of derivatives. This level of toxicity is very similar to that achieved using a complementary pair of anti-saporin BsAbs (Figure 3). Interestingly, however, despite giving similar $IC_{50}$ values, the inhibition of [³H]leucine incorporation achieved with gelonin was never as complete as that obtained with saporin, and even when gelonin was added at the highest concentration the maximum inhibition achieved was only 90%, compared with the 98% inhibition seen with saporin.

Similar results were obtained when gelonin was targeted to Daudi cells via CD38 (Figure 4a); however, the inhibition achieved via CD38 was always less than with CD22. The single anti-CD38 BsAbs, [anti-gel-3 × anti-CD38] and [anti-gel-5 × anti-CD38], and the cocktail of two anti-CD38 BsAbs (anti-gel-3 and anti-gel-5) all have higher $IC_{50}$ values than the corresponding anti-CD22 derivatives and were unable to inhibit completely protein synthesis at higher concentrations of gelonin, with maximum inhibition of 60–70%. The assay was repeated with an extended incubation time before the addition of [³H]leucine, 48 h instead of 24 h, but the anti-CD38 BsAbs still failed to achieve complete inhibition of protein synthesis (results not shown). Using the $IC_{50}$ values alone, the CD38 derivatives are between 2- and 5-fold less toxic than the equivalent CD22 reagents.

The flexibility of the BsAb delivery system makes simultaneous targeting of two surface antigens very straightforward, and in Figure 4b we show how a mixture of two anti-gelonin-specific BsAbs, one directed to CD22 and the other to CD38 ([anti-gel-3 × anti-CD22] + [anti-gel-5 × anti-CD38]), can be used to enhance delivery. Interestingly, with this combination, and despite targeting through CD38, which we have shown is not as efficient as CD22, gelonin toxicity at least matches that obtained with a cocktail of anti-CD22 BsAbs (Figure 3b). Thus when gelonin is co-targeted to CD22 and CD38 its cytotoxic profile assumes that of the CD22 target.

### *Binding of [¹²⁵I]RIP to Daudi cells in the presence of BsAb*

We next compared the number of gelonin and saporin molecules delivered to Daudi cells by the various BsAbs. In these experiments the radiolabelled RIP and BsAb were allowed to bind to cells and then cell-bound and free [¹²⁵I]-RIP were separated by rapid centrifugation of the cells through a mixture of phthalate oils. The binding of [¹²⁵I]RIP to Daudi cells was determined under the same conditions (RIP concentration range and BsAb concentration) as those used in the cytotoxicity assays (Figures 3 and 4). Figure 5a shows the binding curves obtained using a single or a pair of BsAbs to tether [¹²⁵I]gelonin or [¹²⁵I]saporin via CD22. The number of gelonin molecules delivered by the two single anti-gelonin BsAbs, [anti-gel-3 × anti-CD22] and [anti-gel-5 × anti-CD22], was higher than the number of saporin molecules delivered by [anti-sap-1 × anti-CD22], with 15 000 and 23 000 molecules of gelonin delivered at $3 \times 10^{-9}$ M toxin compared with 8000 molecules of saporin. The ability of these single BsAbs to capture radiolabelled RIP correlates closely with their measured binding constants given in Table II. As expected, using a complementary pair of BsAbs in this assay increased the avidity of binding considerably and resulted in approximately 90 000 molecules of [¹²⁵I]gelonin

**Figure 3** Comparison of the cytotoxicity of saporin and gelonin in the presence of anti-CD22 BsAb. Cells ($5 \times 10^5$) were cultured in supplemented RPMI containing gelonin (solid lines) or saporin (dashed lines) at the concentrations shown and BsAb at 1 μg ml⁻¹ for 24 h at 37°C. The wells were then pulsed with 0.5 μCi of [³H]leucine for a further 16 h before havesting the cells and determining the incorporation of radioactive counts. Gelonin alone (●——●); saporin alone (x — x); [anti-gel-2 × anti-CD22] (∇——∇); [anti-gel-3 × anti-CD22] (O——O); [anti-gel-5 × anti-CD22] (□——□); [anti-gel-3 × anti-CD22] + [anti-gel-5 × anti-CD22] (■——■); [anti-sap-1 × anti-CD22] (Δ — Δ); [anti-sap-1 × anti-CD22] + [anti-sap-5 × anti-CD22] (▲ — ▲).

**Table II** Kinetic binding constants for anti-gelonin antibodies

| *Antibody* | $k_{ass}$ ($M^{-1}s^{-1}$) | $k_{diss}$ ($s^{-1}$) | $K_d$ (M) |
|---|---|---|---|
| Anti-gel-2 | $6.02 \pm 0.07 \times 10^4$ | $8.19 \pm 0.69 \times 10^{-4}$ | $1.36 \times 10^{-8}$ |
| Anti-gel-3 | $5.03 \pm 0.15 \times 10^4$ | $3.03 \pm 0.39 \times 10^{-4}$ | $6.02 \times 10^{-9}$ |
| Anti-gel-5 | $5.66 \pm 0.06 \times 10^4$ | $9.25 \pm 1.55 \times 10^{-5}$ | $1.63 \times 10^{-9}$ |
| Anti-sap-1 | $29.20 \pm 1.14 \times 10^4$ | $5.43 \pm 0.26 \times 10^{-3}$ | $1.86 \times 10^{-8}$ |
| Anti-sap-5 | $1.62 \pm 0.02 \times 10^4$ | $2.05 \pm 0.15 \times 10^{-3}$ | $1.27 \times 10^{-7}$ |

The $k_{ass}$ and $k_{diss}$ values of the Fab′ fragments from the anti-gelonin MAbs were determined as shown in Figure 2 using the IAsys resonant mirror biosensor. The values for the anti-saporin MAbs are taken from George *et al.* (1994).

**Figure 4** Comparison of the cytotoxicity of gelonin targeted via CD22 and CD38. Uptake of [$^3$H]leucine by Daudi cells was measured as described in Figure 3. (**a**) Gelonin alone (●——●); [anti-gel-3 x anti-CD38] (O —— O); [anti-gel-5 x anti-CD38] (□ —— □); [anti-gel-3 x anti-CD38] + [anti-gel-5 x anti-CD38] (■ —— ■); [anti-gel-5 x anti-CD22] (□——□); and [anti-gel-3 x anti-CD22] + [anti-gel-5 x anti-CD22] (■——■). (**b**) Gelonin alone (●——●); [anti-gel-5 x anti-CD38] (□ —— □); [anti-gel-3 x anti-CD22] (O——O); and [anti-gel-5 x anti-CD38] + [anti-gel-3 x anti-CD22] (▲——▲).

and 30 000 molecules of [$^{125}$I]saporin binding to each cell at a toxin concentration of $3 \times 10^{-9}$ M.

The results in Figure 5b show similar data for radiolabelled gelonin binding to Daudi cells via anti-CD38 BsAb. In general, CD38-specific BsAbs capture between two and three times more RIP than CD22 BsAb. This difference reflects the increased level of CD38 expression on Daudi cells (unpublished observations). As with the CD22-specific reagents, we obtained a sizeable increase in avidity using a pair of anti-CD38 BsAbs, allowing approximately 250 000 molecules of gelonin to bind to each cell at $3 \times 10^{-9}$ M toxin. Figure 5b also shows that very similar levels of binding were achieved when gelonin was tethered via CD38 alone using a pair of CD38-specific BsAbs, or via CD38 and CD22 using a combination of CD38- and CD22-specific BsAbs.

Using these binding data we were able to estimate, for each BsAb and each combination of BsAbs, the number of gelonin or saporin molecules bound to the target cells at their respective $IC_{50}$ values obtained in toxicity studies (Figures 3 and 4). The results are summarised in Table III. When saporin is targeted via CD22, either with a single BsAb or with a pair of BsAbs, approximately 1000 molecules of saporin will be bound to the cell surface at the $IC_{50}$. In contrast, to achieve an $IC_{50}$ using gelonin, between 6000 and 10 000 molecules are required at the cell surface. Comparing the delivery of gelonin via CD22 and CD38 reveals a striking difference in efficiency between the two target antigens. With anti-CD38 BsAbs, either singly or in pairs, half-maximum inhibition of protein synthesis was achieved only when between 35 000 and 60 000 molecules of gelonin were bound at the cell surface. With the combination of one BsAb directed at CD22 and one BsAb directed at CD38, the efficiency approached that obtained with single or pairs of anti-CD22 BsAbs, with 12 000 molecules of gelonin bound at the $IC_{50}$ concentration.

**Figure 5** Binding of [$^{125}$I]gelonin and [$^{125}$I]saporin to Daudi cells in the presence of anti-CD22- or anti-CD38-specific BsAbs. BsAb (1 μg ml$^{-1}$) was incubated with various concentrations of [$^{125}$I]RIP for 1 h at 37°C. Daudi cells ($5 \times 10^6$) were then added and the incubation continued for a further 1 h. Any endocytosis of cell-bound complexes was inhibited by including 15 mM sodium azide and 50 mM 2-deoxyglucose. The cells were then sedimented through phthalate oils and the pellet counted for radioactivity. The results are expressed as the number of molecules of gelonin or saporin bound per cell. (**a**) Binding via CD22: gelonin + [anti-gel-3 x anti-CD22] (O——O); gelonin + [anti-gel-5 x anti-CD22] (□——□); gelonin + [anti-gel-3 x anti-CD22] + [anti-gel-5 x anti-CD22] (■——■); saporin + [anti-sap-5 x anti-CD22] (Δ —— Δ); and saporin + [anti-sap-1 x anti-CD22] + [anti-sap-5 x anti-CD22] (▲ —— ▲). Results show means ± s.e.m. of three experiments. (**b**) Binding via CD38 and CD38/CD22: gelonin + [anti-gel-3 x anti-CD38] (O —— O); gelonin + [anti-gel-5 x anti-CD38] (□ —— □); gelonin + [anti-gel-3 x anti-CD38] + [anti-gel-5 x anti-CD38] (■ —— ■); and gelonin + [anti-gel-5 x anti-CD38] + [anti-gel-3 x anti-CD22] (▼——▼). Results show average of two experiments.

### *Kinetics of inhibition of [$^3$H]leucine uptake*

The rate at which gelonin and saporin inhibited [$^3$H]leucine uptake into Daudi cells in the presence of BsAbs was examined. A range of concentrations of saporin and gelonin

(0.02–20 μg $ml^{-1}$) were investigated for each single or pair of anti-CD22 BsAbs. The maximum rate of inhibition was achieved when saporin or gelonin was included at a concentration of 2 μg $ml^{-1}$ or above (Figure 6a, inset). In all subsequent experiments RIPs were used at 2 μg $ml^{-1}$. Figure 6a (main figure) shows the rate of inhibition of [$^3$H]leucine incorporation with CD22-specific BsAbs. In all cases there was a lag period of at least 6 h before any inhibition was recorded. When the inhibition of [$^3$H]leucine uptake did commence, saporin was significantly more active than gelonin, achieving 90% inhibition by 24 h. By extrapolation, gelonin would have taken around 40 h to achieve this level of inhibition. Interestingly, the rate of inhibition was the same whether the toxin was delivered by a single BsAb or by a combination of BsAbs.

**Figure 6** The kinetics of gelonin and saporin toxicity in the presence of BsAb. Daudi cells (5 × $10^5$) were incubated with BsAb (1 μg $ml^{-1}$) and gelonin or saporin at the required concentration at 37°C (see below). At selected intervals wells were pulsed with 1 μCi of [$^3$H]leucine for 30 min and the incorporation stopped by the addition of 30 μl of 5 mM cycloheximide + 20 mg $ml^{-1}$ L-leucine. At the end of the time course the cells were harvested and the incorporation of radioactivity determined. (**a**) (gelonin and saporin at 2 μg $ml^{-1}$ throughout) Gelonin + [anti-gel-5 × anti-CD22] (□——□); gelonin + [anti-gel-3 × anti-CD22] + [anti-gel-5 × anti-CD22] (■——■): saporin + [anti-sap-1 × anti-CD22] (△——△); and saporin + [anti-sap-1 × anti-CD22] + [anti-sap-5 × anti-CD22] (▲——▲). Inset: Single BsAb [anti-sap-1 × anti-CD22] and saporin at: I, 0.02 μg $ml^{-1}$; II, 0.2 μg $ml^{-1}$; III, 2 μg $ml^{-1}$; or IV, 10 μg $ml^{-1}$. Similar results were obtained with gelonin. Concentrations above 2 μg $ml^{-1}$ gave a maximum rate of inhibition of [$^3$H]leucine for both saporin and gelonin. (**b**) (gelonin and saporin at 2 μg $ml^{-1}$ throughout) Gelonin + [anti-gel-5 × anti-CD38] (□——□); gelonin + [anti-gel-3 × anti-CD38] + [anti-gel-5 × anti-CD38] (■——■): and gelonin + [anti-gel-5 × anti-CD38] + [anti-gel-3 × anti-CD22] (▼——▼).

When gelonin is delivered via CD38 (Figure 6b), again we see a long lag period before any inhibition of protein synthesis can be measured. This is followed by even slower kinetics for the inhibition of protein synthesis than when gelonin was targeted via CD22, and by extrapolation [$^3$H]leucine uptake would have taken around 60 h to be reduced to 10% of the control level. However, one of the most important findings from this work is that, when gelonin is delivering via CD22 and CD38, using a mixed cocktail of BsAbs, the rate of inhibition increases to that achieved with anti-CD22 BsAbs. Thus, by delivering through two surface antigens, we have increased the activity of the CD38 derivative to that of the anti-CD22 BsAb.

### Discussion

In the current work we have investigated anti-CD22- and anti-CD38-specific BsAbs for the delivery of gelonin against neoplastic B cells. Six new anti-gelonin MAbs were raised by conventional MAb technology and then epitope mapped on gelonin using the IAsys. The IAsys allowed rapid analysis of the binding characteristics of the new MAbs and proved extremely efficient at identifying MAbs which recognised different, non-overlapping epitopes on gelonin. From the panel of MAbs, three (anti-gel-2, -3 and-5) were selected as recognising non-blocking epitopes on gelonin. The $K_d$ of these MAbs ranged from approximately 1 × $10^{-8}$ to 6 × $19^{-9}$ M, with two MAbs, anti-gel-3 anti-gel-5, having respectively three and ten times higher affinity than the best of our anti-saporin MAbs, anti-sap-1 (Table II). Interestingly, one of us (AJTG) has shown that a major difference between these anti-gelonin MAbs and a panel of our anti-saporin MAbs is that in general the latter have strikingly faster off-rates. The results in Table II show that the three anti-gelonin MAbs have $k_{diss}$ values which are between 6 and 50 times slower than anti-sap-1. One possible explanation for this disparity is that during an immune response, because saporin is more toxic than gelonin, most responding B cells may be killed as a result of internalising even a small amount of saporin via their surface Ig. However, those B cells which express surface antibody with a very fast off-rate may engage saporin briefly and achieve activation before the toxin has been carried inside the cell (George *et al*., 1994).

For the current work, Fab' from anti-gel-2, -3 and -5 was constructed into bispecific F(ab')$_2$ antibodies with Fab' from either anti-CD22 or anti-CD38 as their anti-B-cell arm. The three anti-CD22 derivatives performed well in delivering gelonin to Daudi cells and enhanced the toxicity of gelonin between 400- and 2000-fold. As expected, targeting activity showed a strong correlation with the affinity of the anti-gelonin MAbs used in the construction of BsAbs. However, we consistently found that, either as free RIP or when delivered by a BsAb, the gelonin was significantly less toxic than saporin. For example, gelonin delivered by the most effective single BsAb, [anti-gel-5 × anti-CD22], was 10-fold less toxic than saporin delivered by [anti-sap-1 × anti-CD22]. This difference was not due to the BsAb capturing less gelonin on the cell surface, since binding experiments with radiolabelled RIPs showed that the level of gelonin bound by [anti-gel-5 × anti-CD22] was around 3-fold higher than that of saporin bound by [anti-sap-1 × anti-CD22], consistent with anti-gel-5 having a higher affinity than anti-sap-1. By combining the binding data with the results of the cytotoxicity experiments, we found that, for gelonin delivered by a single BsAb, half-maximum inhibition of protein synthesis was not achieved until approximately 6000–10 000 molecules were bound to each cell, while only 1000 molecules of saporin per cell were required to reach this level of toxicity.

Table III Summary of toxicity and binding study using BsAbs against CD22 and CD38 to deliver saporin and gelonin to Daudi cells

| Derivative[a] | $IC_{50}$[b] (M) | Molecules at $IC_{50}$[c] | Fold increase[d] |
|---|---|---|---|
| Saporin alone | $3.6 \pm 1.0 \times 10^{-7}$ | | |
| Gelonin alone | $1.7 \pm 0.9 \times 10^{-6}$ | | |
| Anti-CD22 reagents (saporin) | | | |
| [anti-sap-1 x anti-CD22] | $1.1 \pm 0.2 \times 10^{-10}$ | 1000 | 3600 |
| [anti-sap-1 x anti-CD22] + [anti-sap-5 x anti-CD22] | $1.0 \pm 0.2 \times 10^{-11}$ | 1500 | 36000 |
| Anti-CD22 reagents (gelonin) | | | |
| [anti-gel-2 x anti-CD22] | $4.4 \pm 1.3 \times 10^{-9}$ | ND[e] | 400 |
| [anti-gel-3 x anti-CD22] | $2.3 \pm 0.9 \times 10^{-9}$ | 10000 | 700 |
| [anti-gel-5 x anti-CD22] | $8.2 \pm 0.3 \times 10^{-10}$ | 9000 | 2100 |
| [anti-gel-3 x anti-CD22] + [anti-gel-5 x anti-CD22] | $2.9 \pm 1.3 \times 10^{-11}$ | 6000 | 59000 |
| Anti-CD38 reagents | | | |
| [anti-gel-3 x anti-CD38] | $3.9 \pm 0.1 \times 10^{-9}$ | 50000 | 400 |
| [anti-gel-5 x anti-CD38] | $1.6 \pm 0.2 \times 10^{-9}$ | 35000 | 1100 |
| [anti-gel-3 x anti-CD38] + [anti-gel-5 x anti-CD38] | $1.6 \pm 0.2 \times 10^{-10}$ | 60000 | 10600 |
| Anti-CD22/anti-CD38 cocktail | | | |
| [anti-gel-3 x anti-CD22] + [anti-gel-5 x anti-CD38] | $1.3 \times 10^{-11}$ | 12000 | 130800 |

[a]Antibody derivatives were either single or pairs of bispecific $F(ab')_2$ antibodies as indicated at 1 $\mu g\,ml^{-1}$. The pairs of BsAbs were equal quantities (0.5 $\mu g\,ml^{-1}$) of the two indicated reagents which reacted with gelonin or saporin through two different, non-blocking, epitopes. [b]RIP concentrations giving half-maximum incorporation of [$^3$H]leucine in cytotoxicity experiments (see Figures 3 and 4). Each result shows the mean molar concentration and the standard error obtained from three independent experiments, except for the final result (cocktail of anti-CD22 and anti-CD38 BsAbs), which is the mean $IC_{50}$ obtained from two experiments. [c]Average number of RIP molecules bound per Daudi cell at the $IC_{50}$ concentration taken from the binding studies (Figure 5). [d]Fold increase in gelonin or saporin toxicity when incubated with BsAb as compared with that for gelonin or saporin alone (values given to the nearest 100). [e]Not determined.

Thus as a free molecule and when delivered by a BsAb, gelonin is 5- to 10-fold less toxic than saporin to Daudi cells. An explanation for the difference in toxicity between saporin and gelonin may lie in the finding that, to achieve full inactivation of ribosomes, gelonin requires a co-factor (Carnicelli *et al.*, 1992) identified as RNA (Brigotti *et al.*, 1994), whereas saporin does not. It is possible that the lower toxicity of this RIP is due to a low level of this co-factor in target cells.

Despite this difference in toxicity, when delivered by a complementary pair of BsAbs, gelonin achieved an $IC_{50}$ ($2 \times 10^{-11}$ M) which was effectively equivalent to that given by saporin ($1.5 \times 10^{-11}$ M) (Figure 3 and Table III). Since we have already established that gelonin is less toxic than saporin, the explanation for such potency probably lies in the very high avidity with which the selected pair of anti-gelonin BsAbs captured gelonin at the cell surface. The binding data support this conclusion, showing that, depite their similar $IC_{50}$ values, the pair of anti-gelonin BsAbs are binding approximately six times more RIP to each cell than are the anti-saporin BsAbs. The implications of this result are very important for patient treatment because it shows that with the available mixtures of BsAbs the therapeutic ratio (targeted toxicity/non-specific toxicity) of gelonin is greater than that of saporin. Further studies are under way to confirm this observation.

Previous work has shown that CD22 (Bonardi *et al.*, 1993), CD25 (Tazzari *et al.*, 1993) and CD40 (unpublished observations) are highly effective targets for delivering BsAb–saporin complexes into human neoplastic B cells. We have found that a range of other surface molecules on B cells, such as CD19, CD37 and Ig, were very poor, or completely ineffective, at mediating transport of BsAb–saporin complexes inside cells and augmenting inhibition of protein synthesis (Bonardi *et al.*, 1993). It is now evident that CD38 can also be used to target RIP in this delivery system. However, its performance, while much better than that of CD19 and CD37, is not as good as that of CD22. The $IC_{50}$ values achieved with anti-CD38 BsAbs were 2–10 times higher than with equivalent anti-CD22 reagents, and most importantly the toxicity curves often failed to reach the baseline, showing that the inhibition of protein synthesis was not complete. Binding data strongly suggest that, despite high levels of expression, Daudi cells either internalise CD38 poorly or deliver CD38-bound BsAb–RIP complexes to an inappropriate compartment inside the cell which prevents efficient translocation of RIPs into the cytosol. For example, between 35 000 and 60 000 molecules of gelonin are needed on the surface of each cell to achieve half-maximum inhibition of protein synthesis. These values compare with 6000–10 000 molecules per cell when gelonin is targeted via CD22 (Table III). Similarly, the failure of anti-CD38 derivatives to block protein synthesis completely and the relatively slow kinetics of the inhibition probably reflect poor internalisation relative to CD22.

Perhaps the most interesting finding to emerge from the current work comes from using combinations of BsAbs which engage two distinct cellular targets simultaneously. Using a complementary pair of anti-gelonin BsAbs, one targeting CD22 and the other CD38, we have produced a complex which delivers gelonin to Daudi cells with an efficiency which is close to that achieved by our best CD22 derivatives. Using the most effective pair of anti-CD38 BsAbs, gelonin toxicity could be increased about 11 000 times over that of the free RIP. However, with a mixed pair of BsAbs which target CD22 and CD38 simultaneously, we have increased gelonin toxicity approximately 130 000 times. Thus, this cocktail is delivering gelonin with an efficiency which is equal to that of the pair of anti-CD22 BsAbs. The binding data confirm this intepretation, showing that, while a pair of anti-CD38 BsAbs needs to capture 60 000 gelonin molecules per cell to achieve half-maximum inhibition, the anti-CD22/anti-CD38 BsAbs accomplished this with only 12 000 gelonin molecules per cell, a value which is very similar to that given by the pair of CD22 BsAbs. Thus, by binding CD22 and CD38 simultaneously we appear to gain the advantages of capturing the RIP molecules bivalently and internalising them with the efficiency of CD22. The most likely explanation for this finding is that the anti-CD22 arm of the anti-CD22/anti-CD38/gelonin complex is 'dragging' or 'piggy-backing' the CD38 molecules inside the cells. It may be that the high density of CD38 on the target cells facilitates the initial capture of the complex via its anti-CD38 arm with subsequent binding of the anti-CD22 arm. For future patient therapy, targeting dual antigens in the way described looks very attractive. Antigen density on tumour cells will effectively be increased, and variant cells which fail to express one or other of the target antigens may be susceptible to killing via the second.

We conclude that the targeting system which has been developed for gelonin will complement the saporin system which is currently being evaluated in clinical trials (Bonardi *et al.*, 1992). Because of its relative lack of toxicity, gelonin is clearly an attractive RIP for human treatment and gives a large therapeutic 'window' when targeted with BsAbs. In addition, we anticipate being able to use BsAb–saporin and BsAb–gelonin complexes in combination in individual patients. Thus, when treatment with one RIP results in an anti-RIP response, the RIP will be changed and the period of treatment thereby extended.

**Acknowledgements**
This work has been supported in the UK by Tenovus of Cardiff, The Muriel Edith Rickman Trust and the Cancer Research Campaign, and in Italy by the Ministero dell'Universita, the Consiglio Nazionale delle Richerche and the Associazione Nazionale per la Ricerca sul Cancro. We would like to acknowledge colleagues in the Tenovus laboratory for technical assistance and helpful discussion of the project and John Aherne for his help with the IAsys system.

### References

BARBIERI L, BATTELLI MG AND STIRPE F. (1993). Ribosome-inactivating proteins from plants. *Biochim. Biophys. Acta.*, **1154**, 237–282.

BATTELLI MG, BARBIERI L AND STIRPE F. (1990). Toxicity of, and histological lesions caused by, ribosome inactivating proteins, their IgG conjugates, and their homopolymers. *Acta Pathol. Microbiol. Immunol. Scand.*, **98**, 585–593.

BETTER M, BERNHARD SL, FISHWILD DM, NOLAN PA, BAUER RJ, KUNG AH AND CARROLL SF. (1994). Gelonin analogs with engineered cysteine residues form antibody immunoconjugates with unique properties. *J. Biol. Chem.*, **269**, 9644–9650.

BLAKEY DC AND THORPE PE. (1988). An overview of therapy with immunotoxins containing ricin or its A-chain. *Antibody Immunoconjugates Radiopharmacol.*, **1**, 1–16.

BOLOGNESI A, TAZZARI PL, TASSI C, GROMO G, GOBBI M AND STIRPE F. (1992). A comparison of anti-lymphocyte immunotoxins containing different ribosome inactivating proteins. *Clin. Exp. Immunol.*, **89**, 341–346.

BONARDI MA, BELL A, FRENCH RR, GROMO G, HAMBLIN T, MODENA D, TUTT AL AND GLENNIE MJ. (1992). Initial experience in treating human lymphoma with a combination of bispecific antibody and saporin. *Int. J. Cancer*, **7**, 73–77.

BONARDI MA, FRENCH RR, AMLOT P, GROMO G, MODENA D AND GLENNIE MJ. (1993). Delivery of saporin to human B-cell lymphoma using bispecific antibody: targeting via CD22 but not CD19, CD37, or immunoglobulin results in efficient killing. *Cancer Res.*, **53**, 3015–3021.

BRIGOTTI M, CARNICELLI D, SPERTI S AND MONTANARO L. (1994). RNA present in post-ribosomal supernatants makes ribosomes susceptible to inactivation by gelonin and alpha-sarcin. *Biochem. Mol. Biol. Int.*, **32**, 585–596.

BUCKLE PE, DAVIES RJ, KINNING T, YEUNG D, EDWARDS PR, POLLARD-KNIGHT D AND LOWE CR. (1993). The resonant mirror: a novel optical biosensor for direct sensing of biomolecular interactions. II: Applications. *Biosensors Bioelectronics*, **8**, 355–368.

CARNICELLI D, BRIGOTTI M, MONTANARO L AND SPERTI S. (1992). Differential requirement of ATP and extra-ribosomal proteins for ribosome inactivation by eight RNA N-glycosidases. *Biochem. Biophys. Res. Commun.*, **182**, 579–582.

CUSH R, CRONIN JM, STEWART WJ, MAULE CH, MOLLOY J AND GODDARD NJ. (1993). The resonant mirror: a novel optical biosensor for direct sensing of biomolecular interactions. I. Principles of operation and associated instrumentation. *Biosensors Bioelectronics*, **8**, 347–353.

DOWER SK, DE LISI C, TITUS JA AND SEGAL DM. (1981). Mechanism of binding of multivalent complexes to Fc receptors. I. Equilibrium binding. *Biochemistry*, **20**, 6326–6334.

ELLIOT TJ, GLENNIE MJ, MCBRIDE HM AND STEVENSON GT. (1987). Analysis of the interaction of antibodies with immunoglobulin idiotype of neoplastic B lymphocytes: implications for immunotherapy. *J. Immunol.*, **138**, 981–988.

FAZEKAS DE ST GROTH S AND SCHEIDEGGER D. (1980). Production of monoclonal antibodies: strategies and tactics. *J. Immunol. Methods*, **35**, 1–21.

FRENCH RR, COURTENAY AE, INGAMELLS S, STEVENSON GT AND GLENNIE MJ. (1991). Cooperative mixtures of bispecific $F(ab')_2$ antibodies for delivering saporin to lymphoma *in vitro* and *in vivo*. *Cancer Res.*, **51**, 2353–2361.

GEORGE AJT, FRENCH RR AND GLENNIE MJ. (1994). Measurement of kinetic binding constants of a panel of anti-saporin antibodies using a resoant mirror biosensor. *J. Immunol. Methods* (in press).

GLENNIE MJ, MCBRIDE HM, WORTH AT AND STEVENSON GT. (1987). Preparation and performance of bispecific $F(ab'\gamma)_2$ antibody containing thioether-linked Fab'γ fragments. *J. Immunol.*, **139**, 2367–2375.

GLENNIE MJ, BRENNAND DM, BRYDEN F, MCBRIDE HM, STIRPE F, WORTH AT AND STEVENSON GT. (1988). Bispecific $F(ab'\gamma)_2$ antibody for the delivery of saporin in the treatment of lymphoma. *J. Immunol.*, **141**, 3662–3670.

GLENNIE MJ, TUTT AL AND GREENMAN J. (1993). Preparation of multispecific $F(ab')_2$ and $F(ab')_3$ antibody derivatives. In *Tumour Immunobiology, A Practical Approach*, Gallagher G, Rees RC and Reynolds CW. (eds) pp. 225–244. IRL Press at Oxford University Press: Oxford.

KOHLER G AND MILSTEIN C. (1965). Continuous cultures of fused cells secreting antibody of predefined specificity. *Nature*, **256**, 495–497.

LAMBERT JM, SENTER PD, YAU-YOUNG A, BLATTLER WA AND GOLDMACHER VS. (1985). Purified immunotoxins that are reactive with human lymphoid cells. Monoclonal antibodies conjugated to the ribosome-inactivating proteins gelonin and the pokeweed anti-viral proteins. *J. Biol. Chem.*, **260**, 12035–12041.

MARKWELL MAK. (1982). A new solid state reagent to iodinate protein. I. Conditions for the efficient labelling of anti-serum. *Anal. Biochem.*, **125**, 427–432.

MASON DY, STEIN H, GERDES J, PULFORD KAF, RALFKIAER E, FALINI B, ERBER WN, MICKLEM K AND GATTER KC. (1987). Value of monoclonal anti-CD22 (p135) antibodies for the detection of normal and neoplastic B lymphoid cells. *Blood*, **69**, 836–840.

PELTIER P, CURTET C, CHATAL JF, LE DOUSSAL JM, DANIEL G, AILLET G, GRUAZ-GUYON A, BARBET J AND DELAAGE M. (1993). Radioimmunodetection of medullary thyroid cancer using a bispecific anti-CEA/anti-indium-DTPA antibody and an indium-111-labeled DTPA dimer. *J. Nucl. Med.*, **34**, 1267–1273.

RASO V AND GRIFFIN T. (1981). Hybrid antibodies with dual specificity for the delivery of ricin to immunoglobulin-bearing target cells. *Cancer Res.*, **41**, 2073–2078.

SIVAM G, PEARSON JW, BOHN W, OLDHAM RK, SADOFF JC AND MORGAN Jr AC. (1987). Immunotoxins to a human melanoma-associated antigen: comparison of gelonin with ricin and other A chain conjugates. *Cancer Res.*, **47**, 3169–3173.

STIRPE F, OLSNES S AND PIHL A. (1980). Gelonin, a new inhibitor of protein synthesis, non toxic to intact cells. Isolation, characterization and preparation of cytotoxic conjugates with concanavalin A. *J. Biol. Chem.*, **255**, 6947–6955.

STIRPE F, GASPERI-CAMPANI G, BARBIERI L, FALASCA A, ABBONDANZA A AND STEVENS WA. (1983). Ribosome inactivating proteins from the seeds of *Saponaria officinalis L.* (soapwort), of *Agrostemma githago* L (corn cockle) and of *Asparagus officinalis* (asparagus), and from the latex of *Hura crepitans* L. (sandbox tree). *Biochem. J.*, **216**, 617–625.

TAZZARI PL, ZHANG S, CHEN Q, SFORZINI S, BOLOGNESI A, STIRPE F, MORETTA A AND FERRINI S. (1993). Targeting of saporin to CD25-positive normal and neoplastic lymphocytes by an anti-saporin/anti-CD25 bispecific monoclonal antibody; *in vitro* evaluation. *Br. J. Cancer*, **67**, 1248–1253.

THORPE PE, BROWN ANF, ROSS WCJ, CUMBER AJ, DETRE SI, EDWARDS DC, DAVIES AJS AND STIRPE F. (1981). Cytotoxicity acquired by conjugation of an anti-Thy$_{1.1}$ monoclonal and the ribosome-inactivating protein, gelonin. *Eur. J. Biochem.*, **116**, 447–454.

VITETTA ES, FULTON RJ, MAY RD, TILL M AND UHR JW. (1987). Redesigning nature's poisons to create anti-tumor reagents. *Science*, **238**, 1098–1104.